# Members of the *Sinorhizobium meliloti* ChvI regulon identified by a DNA binding screen

**DOI:** 10.1186/1471-2180-13-132

**Published:** 2013-06-13

**Authors:** Louise Bélanger, Trevor C Charles

**Affiliations:** 1 Department of Biology, University of Waterloo, Waterloo, Ontario N2L 3G1, Canada; 2 Present address: Prevtec microbia inc., Saint-Hyacinthe, Québec, Canada

**Keywords:** Rhizobia, Response regulator, DNA-binding, Exopolysaccharide, Alfalfa nodulation, *chvI* regulon, Signal transduction, Transcriptional regulation, DNA binding assay

## Abstract

**Background:**

The *Sinorhizobium meliloti* ExoS/ChvI two component regulatory system is required for N_2_-fixing symbiosis and exopolysaccharide synthesis. Orthologous systems are present in other Alphaproteobacteria, and in many instances have been shown to be necessary for normal interactions with corresponding eukaryotic hosts. Only a few transcriptional regulation targets have been determined, and as a result there is limited understanding of the mechanisms that are controlled by the system.

**Results:**

In an attempt to better define the members of the regulon, we have applied a simple in vitro electrophoretic screen for DNA fragments that are bound by the ChvI response regulator protein. Several putative transcriptional targets were identified and three were further examined by reporter gene fusion experiments for transcriptional regulation. Two were confirmed to be repressed by ChvI, while one was activated by ChvI.

**Conclusions:**

Our results suggest a role for ChvI as both a direct activator and repressor of transcription. The identities and functions of many of these genes suggest explanations for some aspects of the pleiotropic phenotype of *exoS* and *chvI* mutants. This work paves the way for in depth characterization of the ExoS/ChvI regulon and its potential role in directing bacteria-host relationships.

## Background

Two-component regulatory systems (TCRS) are the most abundant and widespread transcriptional regulators in bacteria, as indicated by the number of instances of the Pfam PF00072 response regulator receiver domain [1]. Bacterial genomes typically contain dozens to hundreds of these systems [2]. Response regulator domains of transcriptional regulatory proteins are phosphorylated by cognate sensor histidine kinase proteins in response to changes in environment or growth conditions [3]. This phosphorylation results in conformational change of the response regulator protein, leading to transcriptional activation or repression. Even with the recognized importance of these systems, very few of them have been characterized with regard to the signal input and the regulatory targets.

The ExoS/ChvI two-component regulatory system, consisting of the membrane-spanning histidine protein kinase ExoS and the response regulator ChvI, is found in alphaproteobacterial genomes. In *Agrobacterium tumefaciens*, the ChvG/ChvI system is vital for plant tumor formation, and mutants are sensitive to acidic pH and detergents [4]. The BvrS/BvrR system of *Brucella abortus* is required for virulence [5] and has a broad impact on cell envelope as well as carbon and nitrogen metabolism [6]. The *Bartonella henselae* BatR/BatS system is also involved in regulating virulence-associated genes [7]. Analysis of a mutant of the ExoS homolog of *Rhizobium leguminosarum* confirmed its requirement for successful nodule invasion and nitrogen fixation [8]. This mutant also had a destabilized outer membrane, associated with reduction of *ropB* expression, as well as increased accumulation of intracellular poly-3-hydroxybutrate (PHB), and reduction in exopolysaccharide production. In all cases studied, ExoS/ChvI TCRS and its orthologs play a role, although not well understood, in the bacterial-host interaction.

*Sinorhizobium meliloti exoS* was first identified through a Tn*5* insertion mutant that resulted in overproduction of exopolysaccharide due to disruption of the membrane-spanning portion of the protein, causing constitutive activation of the kinase activity, thus resulting in constant phosphorylation of ChvI [9]. Null mutants of *exoS* and *chvI* are able to trigger the formation of nodules, but those nodules do not develop normally and they do not fix nitrogen [10]. The mutants do not grow on complex or in liquid media, and cultivation on defined agar-media is challenging, a condition that prompted an early conclusion that *exoS* and *chvI* are essential for *S. meliloti* viability [11]. A *chvI* deletion mutant demonstrated enhanced motility, and reduction in PHB accumulation, the opposite of what was found for a *R. leguminosarum exoS* homolog mutant [12]. Similar to the *R. leguminosarum* mutant [8], alterations in LPS were observed. Also isolated in the Tn*5* screen that yielded the constitutively activated exopolysaccharide overproducing *exoS* mutant was a mutant of *exoR*[9]. Evidence has been provided to suggest a direct interaction of ExoR with ExoS in the periplasm, with ExoR binding contributing to the maintenance of ExoS in an inactive conformation [13]. Furthermore, it has been proposed that cleavage of ExoR is induced by some yet unknown environmental signal during infection of the host plant, and this might modulate its ability to bind ExoS [14], resulting in its activation and regulation of the target genes.

The *exoS* gene is situated within an operon along with *hprK*, part of an incomplete phosphotransferase system (PTS) in Alphaproteobacteria. In *S. meliloti,* HprK is involved in succinate mediated catabolite repression [15]. The establishment of a direct functional or regulatory link between the incomplete PTS and the ExoS/ChvI TCRS has been elusive, partly because the systems have often been studied in isolation.

Given the pleotropic nature of the *exoS* and *chvI* null mutants [10], investigation of gene expression using transcriptomics and proteomics might prove less than satisfactory, as the expression of many genes that are not direct regulatory targets is likely to be altered due to physiological changes in the cell. Indeed transcriptomics have identified hundreds of genes whose expression is affected by the *exoS96*::Tn*5* mutation [16]. Comparison of transcriptomes from two different *chvI* mutant strains (gain-of-function versus reduced-function) narrowed the set of genes regulated by ChvI and subsequently facilitated the identification by gel shift assays of three intergenic regions binding ChvI [17] and the determination of an 11-bp-long putative ChvI binding motif. However, for the majority of genes identified as being differentially expressed in a ChvI dependent manner in that study, including the succinoglycan synthesis genes, no binding to upstream regions could be demonstrated. As an alternative, we applied a method to screen for DNA fragments that were directly bound by the ChvI transcriptional regulator. Analysis of these targets suggests important metabolic pathways affected by ChvI regulation. In return, these new findings directed us to uncover better conditions for cultivation of the loss-of-function *chvI* mutants. Further analyses with reporter gene fusion assays confirmed the direct role of ChvI as a repressor for the rhizobactin and SMc00261 operons. It also confirmed the previously discovered direct activation of the *msbA2* operon by ChvI. Methods developed here to identify ChvI targets have proved to be efficient and could be applied to other response regulators.

## Results

### Application of electrophoretic mobility shift assay to the identification of ChvI-regulated genes

To better understand the role of ChvI as a response regulator, it is necessary to identify genes whose transcription is directly influenced by ChvI. To identify specific DNA sequences from plasmid or genomic DNA for which ChvI might have binding affinity, we adapted a method using the electrophoretic mobility shift principle [18]. The plasmid DNA electrophoretic mobility shift assay (PD.EMSA) and genomic DNA electrophoretic mobility shift assay (GD.EMSA) methods involve incubation of purified DNA-binding protein with fractionated DNA, followed by electrophoresis through a native polyacrylamide gel using sodium boric acid (SB) buffer. In this study, the restriction endonuclease Bsp143I was used for DNA fragmentation. The use of SB buffer, a low conductivity medium, and a 14-cm gel as well as running the gel for 3–6 hours at low voltage, allowed unbound DNA fragments to migrate far from the top of the gel while ChvI-bound fragments remained near the wells (see Additional file 1). Inclusion of EDTA in the buffer resulted in no retardation of electrophoretic mobility suggesting an involvement of the putative Mg^2+^ site for ChvI-DNA interaction (see Additional file 2). The slower migrating bands were excised from the gel, purified, and cloned into pUC18 vector from which the insert DNA could be sequenced from each end to determine the extent of each fragment.

Bsp143I-digested pTC198 plasmid DNA was used to perform PD.EMSA (see Additional file 1). This pUC19 clone contains a 5-kb KpnI-fragment from *S. meliloti* Rm1021 spanning across the entire *chvI-hprK* genomic sequence including the intergenic region between *pckA* and *chvI*[10]. This plasmid was employed to optimize the method with a smaller number of fragments than with genomic DNA, thus providing a better resolution on the gel but also increasing the chances of binding to areas surrounding *chvI* and *exoS* to test for possible autoregulation of ExoS/ChvI. Regulation of the adjacent gene *pckA* by *chvG-chvI* has been previously shown for *A. tumefaciens* using reporter gene fusion assays [19], therefore this experiment was also aimed at testing if *S. meliloti* ChvI could bind upstream of *pckA*.

Following the excision of electrophoretic bands from PD.EMSA of pTC198, DNA fragments were cloned into BamHI-linearized pUC18 and sequenced from both ends. Out of four inserts sequenced, three represent a 176-bp fragment (genomic origin from 48523 to 48699) coding for the region upstream of SMc02753, including its start codon. A single clone contained a 395-bp region spanning the upstream sequence of *chvI* and past the translational start site (genomic origin from 51887 to 52281). These results suggest that ChvI might autoregulate its transcription but most importantly, it shows a direct binding affinity between the ChvI and the upstream sequence of *manXhpr* operon part of the PTS system. The ChvI binding to the 176-bp fragment was also confirmed by performing a gel shift assay using a PCR-amplified DNA fragment from pLB102 and the purified ChvI protein (data not shown). Further delineation of this binding was not performed.

After GD.EMSA, the examination of 27 clones resulted in the identification of a large number of additional potential targets for ChvI regulation (Table 1). Fragments ranged in size from 67 bp to 595 bp. Interestingly, the majority of fragments identified were found to be in predicted coding sequences rather than in intergenic regions. Moreover, ChvI-binding fragments are widely distributed across the genome and are not confined to a particular metabolic pathway. Although no one fragment was identified more than once, two non-contiguous fragments that are part of the same gene (*rhtX*) were independently cloned and sequenced. Fragments from the *exoS-chvI* region were not among the sequenced clones. Reporter gene fusions were used to confirm the *chvI*-regulated transcription of three selected genes (see below). These genes were selected based on their availability from a random fusion library [20], to test a mix of inter and intra fragments and to validate the previously described regulation of the *msbA2* gene cluster.

**Table 1:** DNA fragments recovered from GD.EMSA and genes potentially regulated by ChvI

**Fragment**	**Size**	**Genomic origin**		**Position**	**Gene**	**Function**
	**(bp)**	**From**	**To**	**Inter/intra-genic**		**(location)**
		***Chromosome***				
F1	129	60821	60949	intra	SMc02574 (*hisB*)	probable imidazoleglycerol-phosphate dehydratase
F2	304	654156	654459	intra	SMc02281	
F3	595	1085493	1086087	intra	SMc00051 (*phaA2*)	probable Na(+)/H(+)-antiporter (upstream *mucR*)
F4	152	1183131	1183282	intra	SMc02637	(upstream *lpsL and rkpK*)
F5	139	1220301	1220439	intra	SMc00550	ABC transporter ATP-binding transmembrane protein (upstream *psd* and *pssA*)
F6	142	1260626	1260767	intra	SMc00589	(upstream *gal*)
F7	145	1639710	1639854	intra	SMc02076 (*cls*)	putative cardiolipin synthetase transmembrane protein (downstream *exoR*; upstream *xthA2*)
F8	236	1830765	1831000	intra	SMc00262	putative 3-ketoacyl-CoA thiolase
F9	166	2587012	2587177	intra	SMc02733	
F10	256	2991422	2991677	intra	SMc03993	
F11	128	3117150	3117277	intra	SMc03159 (*metN*)	methionine import ATP-binding protein
F12	288	3303566	3303853	intra	SMc02491	
F13	184	3383057	3383240	inter	SMc03267	putative dipeptidase (upstream ABC transporter)
F14	143	3412878	3413020	inter	SMc03297	
		***pSymB***				
F15	184	1266	1449	inter & intra	SMb21653 (*lacF*)	lactose ABC transporter, permease component
F16	148	43628	43775	inter	SMb20032	
F17	150	132970	133119	inter	SMb20119	putative site-specific recombinase
F18	97	221089	221185	intra	SMb20213	(upstream SMb20214)
F19	67	492546	492612	intra	SMb20478	putative dipeptide ABC transporter permease and ATP-binding protein
F20	143	938297	938439	intra	SMb21188	putative acyltransferase (*msbA2* operon)
F21	141	1091213	1091353	intra & inter	SMb21151 /SMb21552 (*aacC4*)	putative aminoglycoside 6′-N-acetyltransferase
F22	221	1588216	1588436	intra	SMb20574	putative maltodextrin α-D-glucosyltransferase
F23	145	1634582	1634726	intra	SMb20615 (*thiC*)	thiamine biosynthesis protein
		***pSymA***				
F24	220	1186198	1186417	intra	SMa2103	oxidoreductase
F25	212	1278398	1278609	intra	SMa2295	penicillin-binding protein
F26	94	1305222	1305315	intra	SMa2337 (*rhtX*)	rhizobactin transporter
F27	148	1305714	1305861	intra	SMa2337 (*rhtX*)	rhizobactin transporter

### Potential functions of identified ChvI-regulated genes

Genes potentially regulated by ChvI are of diverse function (Table 1). Because DNA fragments binding ChvI are often found within a coding sequence and not in intergenic areas, it is difficult to predict if ChvI acts as an activator of an adjacent gene or a repressor of the gene it binds within. In several cases, such as the rhizobactin gene cluster and the *msbA2* gene cluster, the ChvI-binding fragment is found in the first gene of what is predicted to be an operon. Table 1 lists genes found closest to a ChvI-binding DNA fragment but it is possible in many instances that genes further downstream could be part of the same transcript and also be ChvI-regulated. It is also important to note that the sequenced fragments are a subset of cloned fragments and other ChvI targets likely exist. Using the list of potentially ChvI-regulated genes obtained, we queried databases for functional relationships between targets: MetaCyc [21], KEGG [22] and STRING 8.1 [23]. Based on these analyses, a number of functional linkages may be made between some potential ChvI targets.

Two fragments (F15 and F6) are linked to lactose catabolism. One is found in front of the *lacFGZ1K* gene cluster and the second is found in SMc00589 (a conserved hypothetical protein), about 300 bp upstream of *gal* (Smc00588). The *lacFGZ1K* gene cluster encodes genes for lactose ABC-transporter and a β-galactosidase (E.C. 3.2.1.23). β-D-galactose is degraded through the De Ley-Doudoroff pathway in *S. meliloti*[24,25] and *gal* codes for the galactose dehydrogenase (EC 1.1.1.48) of this pathway.

Two other fragments (F7 and F5) suggest that ChvI is involved in regulating phospholipid biosynthesis. One fragment is found in SMc02076 (*cls*) and another one is found in SMc00550, about 300 bp upstream of *psd* (SMc00551) and followed by *pssA* (SMc00552). Cardiolipin is produced in *S. meliloti* and the only gene coding for a cardiolipin synthetase is *cls*[26]. Interestingly, this gene is located about 1 kb downstream of the *exoS*-associated gene *exoR*. Proteins encoded by *psd* (phosphatidylcholine decarboxylase) and *pssA* (phosphatidylserine synthase) are responsible for the biosynthesis of phosphatidylethanolamine and phosphatidylserine respectively, and both of these phospholipids are also intermediates for phosphatidylcholine biosynthesis [27]. Mutants of these genes exhibit deficiencies in alfalfa symbiosis [27]. Aside from phospholipids synthesis, another link was found between SMc00550 and *msbA2* using STRING 8.1. These two genes are homologs and might have similar functions. The fragment F8 found in SMc00262, a putative 3-ketoacyl-CoA thiolase, followed by SMc00261, a putative fatty-acid-CoA ligase, also suggests regulation of lipid metabolism. These genes are putatively involved in fatty acid β-oxidation.

ChvI was also found to bind fragments from genes involved in peptide and methionine transport. A fragment (F13) belongs to the upstream sequence of SMc03267 and four genes encoding a putative dipeptidase and a putative dipeptide ABC-type transporter. Another fragment (F19) is from SMb20478, part of a gene cluster coding for another dipeptide ABC-transporter. MetN involved in importing methionine also has a fragment of its gene having affinity for ChvI.

**Figure 1:**
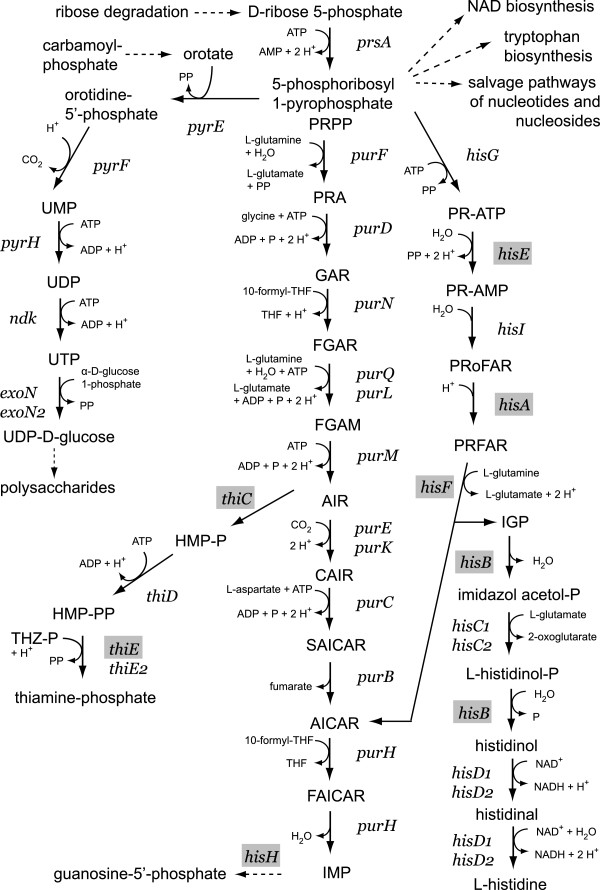
**5-Phosphoribosyl 1-pyrophosphate (PRPP) metabolic pathway and the potential role of ChvI in regulating downstream biosynthesis pathways.** Grey boxes represent genes potentially regulated by ChvI. Uridine-5’-phosphate (UMP), uridine-5’-diphosphate (UDP), uridine-5’-triphosphate (UTP), hydroxymethylpyrimidine phosphate (HMP-P), 4-amino-5-hydroxymethyl-2-methylpyrimidine-pyrophosphate (HMP-PP), 4-methyl-5-(β-hydroxyethyl)thiazole phosphate (THZ-P), 5-phospho-β-D-ribosyl-amine (PRA), 5-phospho-ribosyl-glycineamide (GAR), 5’-phosphoribosyl-N-formylglycineamide (FGAR), 5-phosphoribosyl-N-formylglycineamidine (FGAM), 5-aminoimidazole ribonucleotide (AIR), 4-carboxyaminoimidazole ribonucleotide (CAIR), 5’-phosphoribosyl-4-(N-succinocarboxamide)-5-aminoimidazole (SAICAR), aminoimidazole carboxamide ribonucleotide (AICAR), phosphoribosyl-formamido-carboxamide (FAICAR), inosine-5’-phosphate (IMP), phosphoribosyl-ATP (PR-ATP), phosphoribosyl-AMP (PR-AMP), phosphoribosylformiminoAICAR-P (PRoFAR), phosphoribulosylformimino-AICAR-P (PRFAR), D-erythro-imidazole-glycerol-phosphate (IGP).

A fragment found in *thiC* (F23) and another found in *hisB* (F1) do not present a directly evident link between the thiamine and histidine biosynthesis pathways they are respectively involved in but there is an indirect metabolic link that can be followed in MetaCyc, KEGG and in STRING. ThiC catalyzes the reaction between 5-aminoimidazole ribonucleotide (AIR) and hydroxymethylpyrimidine phosphate (HMP-P) in the thiamine biosynthesis pathway (Figure 1). AIR is biosynthesized from 5-phosphoribosyl 1-pyrophosphate (PRPP). PRPP is also required for the synthesis of histidine. In STRING this link is made through *pur* genes, which code for enzymes involved in purine synthesis. Pyrimidine, purine and pyridine nucleotide synthesis pathways are all dependent on the availability of PRPP.

Following these analyses, we could not find a direct link between these potentially ChvI-regulated genes and the exopolysaccharide biosynthesis pathways, central to one of the most important phenotypes of the *chvI* mutant strain [10]. This is absolutely consistent with other experimental work that has failed to find direct binding of ChvI to exopolysaccharide synthesis gene upstream regions [17]. However, an indirect link is suggested from the regulation of thiamine and histidine biosynthesis (Figure 1). These pathways are inter-related with the synthesis of pyrimidine and consequently the availability of UTP required for the synthesis of UDP-glucose. Perhaps the imbalance caused by deregulating thiamine and histidine synthesis affects UDP-glucose synthesis and therefore polysaccharide production. To test this hypothesis, we added 0.1% uracil to the MM9-succinate minimal media and this improved significantly the growth of the *chvI* mutant strain, although still not to a level comparable to the wild-type (Table 2). However, an important finding from these experiments is that the addition of uracil allows the *chvI* null mutant strain to grow in liquid media. From carbon source utilization analyses performed in a previous work [10], proline or ornithine are good carbon sources for the *chvI* mutant strains, therefore 0.1% proline was added to MM9-succinate media supplemented also with 0.1% uracil. This improved the growth of the mutant strain even further (Table 2).

**Table 2:** **Growth rate constants of *chvI261 *mutant strain grown in MM9-succinate liquid media and with the addition of uracil and/or proline to the growth media**

**Addition to medium**	**Strains**	
	**Rm1021**	**SmUW38**
	**Wild-type**	***chvI261***
none	0.182 ± 0.004	0.043 ± 0.003
uracil	0.167 ± 0.006	0.144 ± 0.004
uracil and proline	0.192 ± 0.003	0.161 ± 0.002
proline	0.201 ± 0.014	0.159 ± 0.025

Errors represent standard deviation.

### Confirmation of ChvI involvement in transcriptional regulation of identified target genes

Having identified genes that might be regulated by ChvI and conditions allowing the growth of the *chvI* mutant strain in liquid media, we used strains from a *S. meliloti* fusion library [20] to confirm the regulation at transcriptional levels. The library had been constructed using a vector that forms gene fusions to the reporter genes *gfp+/lacZ* or *gusA/tdimer2(12)* depending on the orientation of the insert. Because of the possible involvement of ChvI in regulating the *S. meliloti lac* operon, we selected *gusA* fusion strains to measure transcriptional activity using the β-glucuronidase assay. Gene fusions were transduced into *chvI* mutant SmUW38 and into the wild-type strain Rm1021, and then assayed for β-glucuronidase activity and compared. These assays have been applied to three operons identified by the DNA binding assays, confirming the regulation of all three operons by ChvI, and also demonstrating that ChvI can function as either an activator or a repressor, depending on the target gene. The transcription assay with a housekeeping gene in the two genetic backgrounds (wild-type versus *chvI261*) was not tested. However, we did examine expression of the gene SMa2295 with a fusion upstream of the ChvI binding site and the results showed low and not significant GusA activity difference between the two genotype backgrounds (23 versus 30 Miller Units).

**Figure 2:**
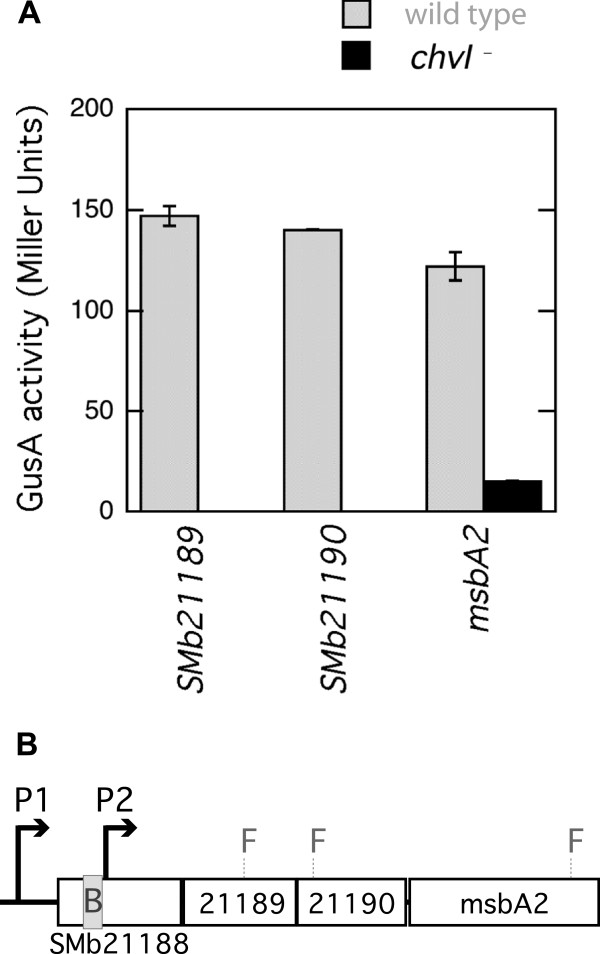
**Transcriptional fusion assays and the *msbA2 *operon.** (**A**) GusA activities were measured for fusions in genes SMb21189, SMb21190, and *msbA2* in wild-type (Rm1021) and *chvI261* mutant (SmUW38) strain backgrounds. No GusA activities above background levels were detected for fusions to SMb21189 and SMb21190 in the *chvI261* mutant strain background. (**B**) In the operon diagram, F1, F2, and F3 represent the positions of the fusions to SMb21189, SMb21190 and *msbA2* respectively. The grey box (**B**) represents the region for ChvI binding, and P1 and P2 are predicted promoters.

ChvI-bound fragment F20 was identified within SMb21188, the first gene of a predicted four-gene operon, and therefore we tested three gene fusions to SMb21189, SMb21190, and *msbA2* (SMb21191) (Figure 2B). These fusions had a much higher expression level in the wild-type than in *chvI* mutant background (Figure 2A). These results suggest that ChvI is responsible for activation of the co-transcription of SMb21189, SMb21190, and *msbA2* genes. Using a neural network promoter prediction tool [28], we predicted a putative transcriptional start site (P2) adjacent to the area containing a ChvI binding site (B). Another putative transcriptional start site (P1) further upstream from SMb21188 suggests that transcription might be directed from two differently regulated promoters, only one of which would include the SMb21188 gene.

**Figure 3:**
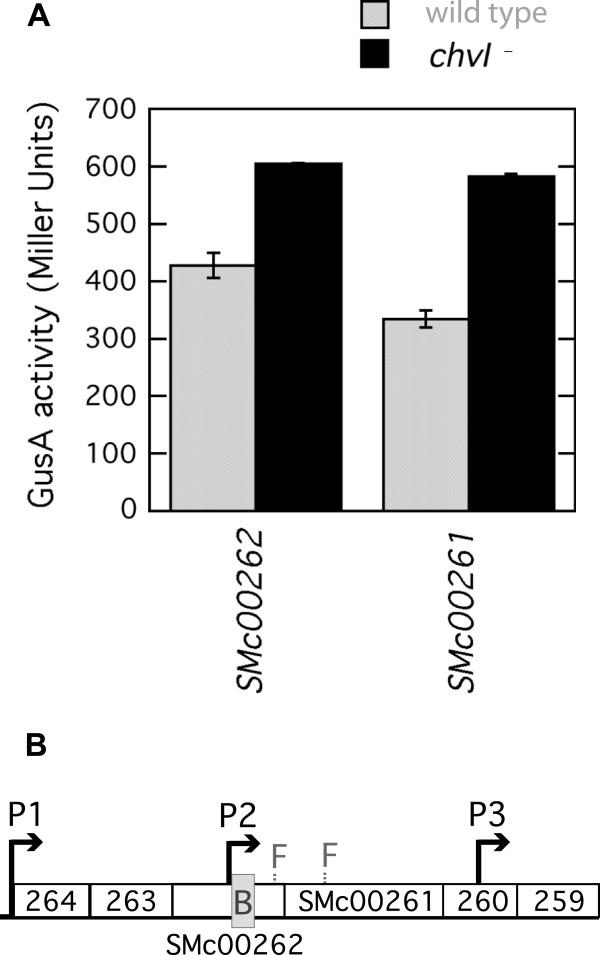
**Transcriptional fusion assays and the SMc00261 operon.** (**A**) GusA activities were measured for fusions in genes SMc00262 and SMc00261 in wild-type (Rm1021) and *chvI261* mutant (SmUW38) strain backgrounds. (**B**) In the operon diagram, F1 and F2 represent the position of the fusions to SMc00262 and SMc00261 respectively. The grey box (**B**) represents the region for ChvI binding, and P1, P2 and P3 are predicted promoters.

Reporter gene fusion assays and promoter prediction were done with fusions in genes SMc00262 and SMc00261, which are predicted to encode a 3-ketoacyl-CoA thiolase and a fatty-acid-CoA ligase respectively (Figure 3B). In this case, a promoter was predicted immediately upstream of the ChvI binding area in SMc00262 and accordingly the fusions further downstream in SMc00262 and in SMc00261 presented higher expression levels in *chvI* mutant strains than in wild type (Figure 3A). These results suggest that ChvI acts by repressing the transcription of the SMc00264 – SMc00259 operon.

**Figure 4:**
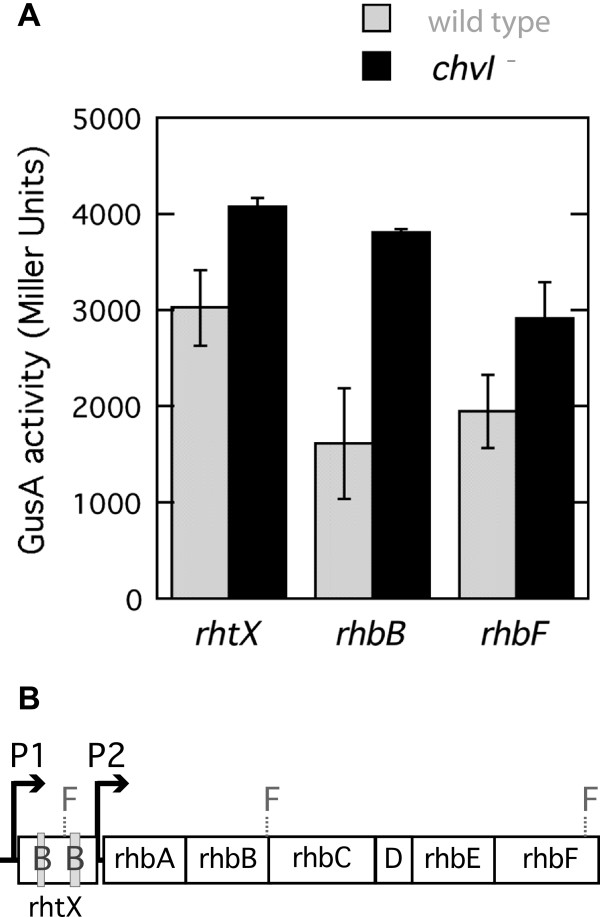
**Transcriptional fusion assays and the rhizobactin operon.** (**A**) GusA activities were measured for fusions in genes *rhtX*, *rhbB* and *rhbF* in wild-type (Rm1021) and *chvI261* mutant (SmUW38) strain backgrounds. (**B**) The rhizobactin genes are clustered in one operon, F1 F2 and F3 represent the positions of the fusions to *rhtX*, *rhtB*, and *rhbF* respectively. The grey boxes (B1 and B2) represent the possible position for ChvI binding, and P1 and P2 are predicted promoters.

*S. meliloti* produces an iron-siderophore, rhizobactin 1021, under iron-depleted conditions [29]. Genes for the synthesis and transport of rhizobactin are clustered in an operon [30]. The rhizobactin transporter coding sequence (*rhtX*, SMa2337) was found to contain two DNA fragments binding ChvI (Table 1 and Figure 4B). We tested a fusion following the first binding site (B1) and two other fusions further in *rhbB* (SMa2402; diaminobutyrate decarboxylase, EC 4.1.1.86) and in *rhbF* (SMa2410). The promoter prediction suggests the presence of a promoter before *rhtX* and another one before *rhbA*. The β-glucuronidase assays presented a higher expression in *chvI* background for all three fusions. This suggests that ChvI represses the expression of genes required for the synthesis and transport of rhizobactin 1021. Both binding areas seem to be important in repressing the transcription as shown by a higher expression in the fusion found before the second binding.

The high basal level of the negatively regulated operons is not really unexpected given that we do not know the repressing conditions, and also the likelihood of multiple regulatory systems acting on these genes. These experiments involved the comparison of gene expression in genetic backgrounds that resulted in differences only in the presence / absence of the ChvI regulator. Otherwise, the environmental conditions were not altered.

## Discussion

An adaptation of methods to perform gel electrophoresis mobility shift assays allowed us to identify DNA fragments with higher affinity for ChvI. Analyses of these results force us to revise our earlier perceptions following phenotypic analyses of ExoS/ChvI as mainly a regulatory system for exopolysaccharide production. Our results suggest that the ChvI regulon includes genes from diverse pathways. Moreover, ChvI appears to have a dual regulatory role, activating and repressing different operons. The total number of targets likely far outnumbers the 27 fragments that we pulled out in our screen, especially considering that we did not hit the same fragment more than once, and we also did not find a few other targets that had previously been shown to be bound by ChvI.

The approach used in our study is highly complementary to the microarray and directed DNA binding study of Chen et al. [17] that resulted in the identification of several potential regulatory targets of ExoS/ChvI and the prediction of a consensus binding sequence. It is important to note, however, that of 19 upstream regions tested, binding was only detected to three (*ropB1*, SMb21440, SMc01580), and a putative consensus sequence was determined using some upstream regions to which binding had not been demonstrated. Confirmation of this consensus binding sequence awaits more detailed DNA footprinting experiments on a larger number of identified targets. It is possible that many ChvI-repressed genes may not have been detected in that study due to the use of a constitutively activated variant of the ChvI protein that might not have been able to function as a repressor.

The binding of ChvI within SMa2337 (*rhtX*) to repress *rhtXrhbABCDEF* gene transcription could suggest that following the sensing of a signal other than the presence of iron, ExoS/ChvI represses genes for rhizobactin 1021 production. This operon is known to be upregulated by RhrA in iron-depleted conditions [31] and downregulated by RirA in iron-replete conditions [32]. Our fusion assay results confirmed that the rhizobactin operon is highly expressed in M9-minimal media (iron-depleted conditions). However, this expression is even higher in strains with the *chvI* null mutation. Iron is an important micronutrient found in many cofactors required for cytochrome and nitrogenase activity. Its acquisition however is difficult for two main reasons. First, it is poorly soluble at pH 7, and secondly, a high concentration of iron can cause the generation of hydroxy radicals. Bacteria produce siderophores to scavenge iron and therefore control iron availability. A tight control over the production of siderophore is thus important. The lack or the overproduction of rhizobactin 1021 by *S. meliloti* impairs the symbiotic relationship with alfalfa [29]. Mutation of *rirA* derepresses rhizobactin production and as a result causes a growth defect of the strain relative to the presence of iron [33]. The reduced viability of the *rirA* mutant due to oxidative stress suggested that perhaps this strain would also be affected in its symbiotic properties but it was not the case [33]. This study suggested that in planta another unknown regulatory system might control the production of rhizobactin. Whether ExoS/ChvI might be the system responsible awaits further investigation.

Another important finding is the confirmation that ChvI is involved in activation of the expression of SMb21189, SMb21190, and *msbA2*. These genes have only been described recently in the literature although *msbA2* in particular may play an important but incompletely defined role in symbiosis [34,35], and the operon has already been shown to be subject to ChvI regulation [17]. SMb21189 and SMb21190 encode glycosyltransferases and *msbA2* is part of an ABC-transporter family involved in macromolecule export. The above mentioned recent studies proposed that the operon including SMb21188, a putative acyltransferase, is involved in the production and export of an unknown polysaccharide which uses intermediates from the succinoglycan production pathway. The regulation of this operon by ExoS/ChvI is therefore the closest link to the succinoglycan-deficient phenotype of *exoS* and *chvI* mutant strains. Although this ChvI-regulated operon is not required for succinoglycan production it seems to be functionally related to succinoglycan production.

The third operon that we confirmed to be differentially regulated by ChvI encodes proteins putatively involved in fatty acid β-oxidation. SMc00262 putatively produces a 3-ketoacyl-CoA and SMc00261, a fatty-acid-CoA ligase. These genes are also followed by SMc00260 coding for a putative short-chain dehydrogenase and SMc00259 coding for a hypothetical protein. Upstream of these genes lay genes for a transcriptional regulator of the IclR family (SMc00263) and another short-chain dehydrogenase (SMc00264). Our earlier studies failed to demonstrate a phenotype for SMc00260 and SMc00264 mutants [36]. A tripartite ATP-independent periplasmic (TRAP) transporter system upregulated by the presence of acetoacetate and 3-methyl oxovaleric acid is encoded by genes in the same orientation upstream of SMc00264 [37]. All these genes are organized in the same orientation and close enough to each other to be part of the same transcript. However, our finding of a ChvI binding site in SMc00262, after the gene encoding the IclR regulator, suggests a complex regulation of these genes. In fact, a N-Acyl homoserine lactone (AHL) also impacts on their expression [38]. The fatty-acid-CoA ligase (SMc00261) has been found differentially accumulated in early log phase cultures of *S. meliloti* Rm1021 treated for 2 hours with 3-oxo-C16:1-HL while the periplasmic binding protein (SMc00265) accumulated in stationary phase cultures independently of the presence of AHLs. Perhaps under conditions that activate ChvI, the first part of the gene cluster is upregulated to allow the import of an organic acid but the second part responsible for its degradation and entry in the TCA cycle is downregulated. This hypothesis would suggest the use of this organic acid, under certain conditions, as a readily available building block rather than an energy source.

An important finding from this work is that uracil and proline improved the growth of the *chvI* mutant. This finding now allows us to culture the mutant strain in liquid media, greatly facilitating experimental analysis. Binding of ChvI in *thiC* (SMb20615) and in *hisB* (SMc02574), perhaps to repress the thiamine and histidine biosynthesis operons, made us hypothesize that a derepression of these operons in *exoS* or *chvI* mutants could lead to a deficiency in UTP formation and could explain the pleiotropy of these mutants. Rhizobial purine and pyrimidine auxotrophic mutants have been found affected in polysaccharides synthesis and plant invasion [39-42]. Further work needs to be done to confirm that *chvI* mutant auxotrophy is truly caused by a derepression of operons for thiamine and histidine biosynthesis.

## Conclusions

We have identified a number of putative direct targets of ChvI, many of which are consistent with the pleotropic phenotype of *exoS* and *chvI* mutants. We also demonstrated that ChvI may act as a repressor or activator of gene expression, and surprisingly ChvI seems to often bind within predicted protein coding sequences. The bias is often to only consider intergenic regions for locations of potential regulatory sites. However, we note that the Fur regulator of *Helicobacter pylori* is just one example of a transcriptional regulatory protein that has targets within polycistronic operons and acts as a repressor and an activator of gene expression [43]. The tendency to search for transcriptional cis-regulatory elements in intergenic areas rather than considering equally regions internal to ORFs may need to be revisited. GD.EMSA or Chromatin-Immunoprecipitation (ChIP) techniques are examples of techniques that do not have a bias towards intergenic or intragenic areas and their usage certainly make important contributions to our knowledge about transcriptional regulation.

Although this study has uncovered new facets of the ExoS/ChvI regulation, the regulatory signal is still unknown. However, a number of new hypotheses emerge. Several genes identified in this study as possibly ChvI-regulated are involved in amino acid and peptide metabolism and transport. In *Rhizobium leguminosarum* bv. *viciae* VF39SM, peptides have been shown to increase the expression of the outer membrane protein *ropB* in a ChvG-dependent manner [44]. Perhaps ExoS and/or ExoR sense(s) peptides. Also, our work suggests a direct regulatory link between the PTS and the ExoS/ChvI systems; do these systems partner to coordinate the C and N metabolism as suggested by recent work in *B. melitensis*[45]? With several putative ChvI-targeted genes now identified, tools necessary to test these hypotheses are available. We are cognizant, however, of the fact that our screen was not saturating, and we will endeavor to adapt the method for higher throughput so that we have a better understanding of the complete ChvI regulon and the nature of the ChvI consensus binding sequence.

## Methods

### Bacterial strains, plasmids and growth conditions

Strains and plasmids used in this study are listed in Table 3. Growth conditions are as described previously [10] or as described in subsequent procedures.

**Table 3:** Bacterial strains, plasmids and primers used in this study

**Strain/Plasmid/Primer**	**Relevant characteristics**	**Reference or source**
*Sinorhizobium meliloti*		
Rm1021	SU47 *str-21*, Sm^r^ wild type	[46,47]
SmUW38	Rm1021 *chvI*::*nptII* (*chvI261*)	[10]
SmFL430	RmP110 SMa2295::pTH1522	[20]
SmFL112	RmP110 *msbA2*::pTH1522	[20]
SmFL4665	RmP110 SMb21189::pTH1522	[20]
SmFL5401	RmP110 SMb21190::pTH1522	[20]
SmFL918	RmP110 SMc00262::pTH1522	[20]
SmFL4392	RmP110 SMc00261::pTH1522	[20]
SmFL2950	RmP110 *rhbB*::pTH1522	[20]
SmFL5628	RmP110 *rhtX*::pTH1522	[20]
SmFL5755	RmP110 *rhbF*::pTH1522	[20]
SmUW45	Rm1021 SMa2295::pTH1522	This study
SmUW43	Rm1021 *msbA2*::pTH1522	This study
SmUW58	Rm1021 SMb21189::pTH1522	This study
SmUW59	Rm1021 SMb21190::pTH1522	This study
SmUW46	Rm1021 SMc00262::pTH1522	This study
SmUW57	Rm1021 SMc00261::pTH1522	This study
SmUW55	Rm1021 *rhbB*::pTH1522	This study
SmUW62	Rm1021 *rhtX*::pTH1522	This study
SmUW63	Rm1021 *rhbF*::pTH1522	This study
SmUW157	SmUW38 SMa2295::pTH1522	This study
SmUW133	SmUW38 *msbA2*::pTH1522	This study
SmUW148	SmUW38 SMb21189::pTH1522	This study
SmUW149	SmUW38 SMb21190::pTH1522	This study
SmUW136	SmUW38 SMc00262::pTH1522	This study
SmUW147	SmUW38 SMc00261::pTH1522	This study
SmUW145	SmUW38 *rhbB*::pTH1522	This study
SmUW152	SmUW38 *rhtX*::pTH1522	This study
SmUW153	SmUW38 *rhbF*::pTH1522	This study
*Escherichia coli*		
DH5α	F^-^, φ80d*lac*Z∆M15, *end*A1, *rec*A1∆(*lac*ZYA-*arg*F)U169, *hsd*R17(r_K_^-^ m_K_^+^), *deo*R, thi-1, *sup*E44, λ^-^, *gyr*A96, *rel*A1	[48]
BL21(DE3)pLysS	F^−^, *ompT*, *hsdS*_B_ (r_B_^-^, m_B_^-^), *dcm*, *gal*, λ(DE3), pLysS, Cm^r^	[49]
***Plasmid***		
pGEM®-T Easy	Cloning of PCR products, Amp^r^	Promega (USA)
pET-30a(+)	His•Tag expression vector, Km^r^	EMD Chemicals (Novagen)
pLB010	pGEM®-T Easy::*chvI*, Amp^r^	This study
pJF011	pET-30a(+)::*chvI*, Km^r^	This study
pUC18	Cloning vector, Amp^r^	[50]
pTC198	pUC19::5-kb *chvI-exoShrpK*, Amp^r^	[10]
pTC190	pVK101::4kb *A. tumefaciens chvI/G*, Tc^r^ Km^r^	[4]
pKNG101	*sacB*^+^*mob*RK2 *ori*_R6K_, Sm^r^	[51]
pKD001	pTC190::pKNG101, Tc^r^	This study
***Primer***	***Sequence (*5′-3′*)***	
LB5	atgcagaccatcgcgctt	This study
LB6	acatcgtgatccaacaagg	This study
LB61	gtaaaacgacggccagt	This study

### *Cloning of* chvI *for His•Tag-ChvI expression and purification*

*S. meliloti* Rm1021 *chvI* was PCR amplified using primers LB5 and LB6 (Table 3). The 800-bp PCR fragment was gel-purified and then cloned in pGEM®-T Easy vector. Plasmid pLB010 with the insert in the correct orientation for expression was verified by DNA sequence analysis. NotI *chvI*-containing fragment was then cut out of pLB010 and ligated to NotI-digested pET-30a, generating a N-terminal His•Tag fusion pJF011. *E. coli* BL21(DE3)pLysS clones carrying the pJF011 plasmid were confirmed for His•Tag-ChvI production by western blot using a His•Tag monoclonal antibody from mouse (Novagen) and Alexa Fluor 488 goat anti-mouse IgG (H + L) (Invitrogen, Molecular Probes) as the secondary antibody. His•Tag-ChvI purification using nickel-affinity chromatography was performed in the laboratory of Professor Bi-Cheng Wang at University of Georgia (USA).

### Electrophoretic mobility shift assay using genomic DNA (GD.EMSA)

To prepare samples, *S. meliloti* Rm1021 genomic DNA was digested to completion by overnight incubation with Bsp143I restriction enzyme (Sau3AI isoschizomer, Fermentas Life Sciences, Canada) and the reaction was then heat-inactivated. Purified His•Tag-ChvI protein was mixed with digested DNA in a solution of 9% glycerol, 3 mM acetyl phosphate, 0.8 mM Tris-acetate, 0.25 mM magnesium acetate, 1.65 mM potassium acetate, 2.5 μg ml^-1^ bovine serum albumin (BSA). For negative controls, ChvI protein was not added to samples. Incubations were carried out for 30 minutes at room temperature and loaded directly on gel without dye.

To perform the electrophoresis, a sodium boric acid buffer (SB buffer) was made following the specifications of Brody and Kern [52]. 5% nondenaturing polyacrylamide gels (14 cm × 16 cm) were cast using a Hoefer SE 600 gel electrophoresis unit and following the standard procedure for resolution of small DNA fragments [53] but using SB buffer instead of TBE buffer. Gels were run in 1X SB buffer between 25 to 40 mA for 3–6 hours. Gels were then stained for 1 hour in a 3X GelRed™ staining solution containing 0.1 M NaCl and following manufacturer’s recommendation for post gel staining (Biotium, USA, CA) prior to visualization on a UV transilluminator. Shifted DNA bands in the highest part of the gel were then excised and stored in 2-ml plastic tubes at −20°C.

To recover DNA fragments from polyacrylamide gel, the method from Ausubel et *al*. (1992) [53] was used. The elution buffer used contained 0.5 M ammonium acetate, 1 mM EDTA, 0.1% SDS and final pH 8. 200 μl elution buffer was added to each tube containing a piece of gel. The gel was then crushed in smaller pieces using a pipet tip. Tubes were incubated overnight at 37°C with shaking. Following centrifugation in a microcentrifuge at room temperature for 10 minutes at 10,000 rpm, supernatant was removed and transferred to a clean 2.0 ml tube. Ethanol (500 μl) was added to precipitate the DNA and tubes were placed at −20°C overnight. DNA was pelleted at 13,000 rpm for 10 minutes. Supernatant was removed and DNA solubilized in 100 μl of 10 mM Tris pH 8 and 15 μl of 5 M sodium chloride was added. DNA was then precipitated a second time with 2 volumes of ethanol and kept overnight at −20°C. Precipitated DNA was recovered by centrifugation in a microcentrifuge at 13,000 rpm for 15 minutes, supernatant was removed and DNA was dried. Final resuspension of DNA was done with 10 μl of 10 mM Tris pH 8.

The DNA fragments were cloned into the BamHI site in pUC18. Prior to ligation, BamHI-digested pUC18 was dephosphorylated using shrimp alkaline phosphatase (Fermentas Inc.) and the reaction stopped by heat-inactivation. Ligation was performed overnight at room temperature with T4 DNA ligase (Fermentas Inc.). Transformation of calcium chloride competent *E. coli* DH5α cells was done following standard procedure [54]. Over 40 transformant colonies were streak-purified from each experiment. A selection of them were then used for plasmid preparation and tested for the presence of an insert using restriction digest with EcoRI and PstI. Fragments cloned in pUC18 were sequenced using primers M13F provided by the sequencing facility (University of Waterloo) or LB61 (Table 3).

Sequences were first analyzed by searching for Sau3AI (Bsp143I) restriction sites to determine the limits of each fragment. Each fragment sequence was then searched against *S. meliloti* Rm1021 genomic sequence using the BLAST tool from Toulouse annotation website [55]. Genes in closest proximity to identified sequences and potentially regulated by ChvI were searched against STRING 8.1 databases (June 28, 2009) for functional relations [23]. The search was directed from the Toulouse annotation website.

### Reporter gene fusion strains

Transcriptional fusion strains were obtained by transduction from the reporter gene fusion library strains made by Cowie *et al*. [20]. SmFL strains were used to prepare transduction lysates to transfer the gene fusions from the original *S. meliloti* RmP110 background into the Rm1021 background. Selection of transductants was done on LB with gentamicin (60 μg ml^-1^). The same lysates were also used to transduce gene fusions into SmUW38 (pKD001) with selection on LB gentamicin (60 μg ml^-1^) and neomycin (200 μg ml^-1^). Four transductants per transduction experiment were picked and streaked on LB gentamicin and neomycin. Transductants were then cured of pKD001 by streaking them on MM9-succinate gentamicin (20 μg ml^-1^) containing 2.5% sucrose and incubated at 30°C for four days. pDK001-cured strains were finally streaked on MM9-succinate gentamicin.

Phage ΦM12 was used for transductions following the usual procedure [56], except that TY media was used instead of LBmc media to prepare and dilute lysates. High yield of transductants required the use of Bacto™-Agar, -Tryptone, and -Yeast extract (BD). Diluted lysate (0.5 ml) was mixed with equal volume of cell suspension and incubated at room temperature for 30 minutes. Cells were then recovered by centrifugation in a microcentrifuge for 10 minutes and washed twice with 2 ml of saline. Final resuspension was done with 400 μl saline and then spread on two agar plates. Plates were incubated at 30°C for four days.

### Growth in liquid media

Inocula were prepared by resuspending bacterial biomass from MM9-succinate-agar plates into a saline solution (0.85% NaCl) to obtain an optical density (OD_600_) of 0.8. Test tubes containing 5-ml liquid media made of MM9-succinate with/without 0.1% proline and/or 0.1% uracil where inoculated with the inoculum at a 10% concentration. Test tubes were incubated at 30°C with constant shaking. Growth was monitored by reading the absorbance at 600 nm. Growth rate constants (μ) were calculated based on absorbance values during the exponential growth phase and using the formula: μ = ( (log_10_ N - log_10_ N_0_) 2.303) / (t - t_0_). Results represent the average of duplicates and the standard deviation was calculated as the error.

### β-Glucuronidase assay

To measure transcription from reporter gene fusion strains, the β-glucuronidase assay described in Cowie et al. [20] was adapted. Strains were grown in MM9-succinate plus 0.1% proline, 0.1% uracil, and gentamicin until OD_600_ of 0.2 - 0.8. These cells were then used directly for the assay in microplates as described previously [20]. Assays were done in triplicate and standard deviation calculated.

## Competing interests

The authors declare that they have no competing interest.

## Authors’ contributions

LB planned and carried out experiments, performed data analysis, and wrote the manuscript. TCC planned experiments and wrote the manuscript. Both authors read and approved the final manuscript.

## Supplementary Material

Additional file 1 **Gel image of PD.EMSA to compare DNA shifts on 6-cm versus 14-cm 5% nondenaturing polyacrylamide gel and using SB buffer.** Prior to the electrophoresis, the Bsp143I restricted pTC198 plasmid was incubated or not with the HisTag-ChvI protein. Click here for file

Additional file 2 **Gel image of PD.EMSA to compare ChvI binding specificity in presence of EDTA or acetylphosphate.** A 5% nondenaturing polyacrylamide gel made with TB buffer was used for the electrophoresis of the EcoRI-PstI double restricted pLB102 plasmid. The plasmid DNA was incubated or not with HisTag-ChvI protein in presence or not of EDTA and in presence or not of acetylphosphate (AP) prior to the electrophoresis. Click here for file

## References

[B1] Finn RD Mistry J Tate J Coggill P Heger A Pollington JE Gavin OL Gunasekaran P Ceric G Forslund K Holm L Sonnhammer ELL Eddy SR Bateman A The Pfam protein families database Nucleic Acids Res 2010 38 D211 D222 10.1093/nar/gkp985 19920124 PMC2808889

[B2] Galperin MY Structural classification of bacterial response regulators: diversity of output domains and domain combinations J Bacteriol 2006 188 4169 4182 10.1128/JB.01887-05 16740923 PMC1482966

[B3] Gao R Stock AM Biological insights from structures of two-component proteins Annu Rev Microbiol 2009 63 133 154 10.1146/annurev.micro.091208.073214 19575571 PMC3645274

[B4] Charles TC Nester EW A chromosomally encoded two-component sensory transduction system is required for virulence of *Agrobacterium tumefaciens* J Bacteriol 1993 175 6614 6625 8407839 10.1128/jb.175.20.6614-6625.1993 PMC206773

[B5] Sola-Landa A Pizarro-Cerdá J Grilló MJ Moreno E Moriyón I Blasco JM Gorvel JP López-Goñi I A two-component regulatory system playing a critical role in plant pathogens and endosymbionts is present in *Brucella abortus* and controls cell invasion and virulence Mol Microbiol 1998 29 125 138 10.1046/j.1365-2958.1998.00913.x 9701808

[B6] Viadas C Rodríguez MC Sangari FJ Gorvel JP García-Lobo JM López-Goñi I Transcriptome analysis of the *Brucella abortus* BvrR/BvrS two-component regulatory system PLoS One 2010 5 e10216 10.1371/journal.pone.0010216 20422049 PMC2858072

[B7] Quebatte M Dehio M Tropel D Basler A Toller I Raddatz G Engel P Huser S Schein H Lindroos HL Andersson SGE Dehio C The BatR/BatS two-component regulatory system controls the adaptive response of *Bartonella henselae* during human endothelial cell infection J Bacteriol 2010 192 3352 3367 10.1128/JB.01676-09 20418395 PMC2897681

[B8] Vanderlinde EM Yost CK Mutation of the sensor kinase *chvG* in *Rhizobium leguminosarum* negatively impacts cellular metabolism, outer membrane stability, and symbiosis J Bacteriol 2012 194 768 777 10.1128/JB.06357-11 22155778 PMC3272964

[B9] Cheng HP Walker GC Succinoglycan production by *Rhizobium meliloti* is regulated through the ExoS-ChvI two-component regulatory system J Bacteriol 1998 180 20 26 9422587 10.1128/jb.180.1.20-26.1998 PMC106843

[B10] Bélanger L Dimmick KA Fleming JS Charles TC Null mutations in *Sinorhizobium meliloti exoS* and *chvI* demonstrate the importance of this two-component regulatory system for symbiosis Mol Microbiol 2009 74 1223 1237 10.1111/j.1365-2958.2009.06931.x 19843226

[B11] Osterås M Stanley J Finan TM Identification of *Rhizobium*-specific intergenic mosaic elements within an essential two-component regulatory system of *Rhizobium* species J Bacteriol 1995 177 5485 5494 7559334 10.1128/jb.177.19.5485-5494.1995 PMC177356

[B12] Wang C Kemp J Da Fonseca IO Equi RC Sheng X Charles TC Sobral BWS *Sinorhizobium meliloti* 1021 loss-of-function deletion mutation in *chvI* and its phenotypic characteristics Mol Plant Microbe Interact 2010 23 153 160 10.1094/MPMI-23-2-0153 20064059

[B13] Chen EJ Sabio EA Long SR The periplasmic regulator ExoR inhibits ExoS/ChvI two-component signalling in *Sinorhizobium meliloti* Mol Microbiol 2008 69 1290 1303 10.1111/j.1365-2958.2008.06362.x 18631237 PMC2652646

[B14] Lu H-Y Luo L Yang M-H Cheng H-P *Sinorhizobium meliloti* ExoR is the target of periplasmic proteolysis J Bacteriol 2012 194 4029 4040 10.1128/JB.00313-12 22636773 PMC3416547

[B15] Pinedo CA Gage DJ HPrK regulates succinate-mediated catabolite repression in the gram-negative symbiont *Sinorhizobium meliloti* J Bacteriol 2009 191 298 309 10.1128/JB.01115-08 18931135 PMC2612420

[B16] Wells DH Chen EJ Fisher RF Long SR ExoR is genetically coupled to the ExoS-ChvI two-component system and located in the periplasm of *Sinorhizobium meliloti* Mol Microbiol 2007 64 647 664 10.1111/j.1365-2958.2007.05680.x 17462014

[B17] Chen E Fisher R Perovich V Sabio E Long S Identification of direct transcriptional target genes of ExoS/ChvI two-component signaling in *Sinorhizobium meliloti* J Bacteriol 2009 191 6833 6842 10.1128/JB.00734-09 19749054 PMC2772461

[B18] Garner MM Revzin A A gel electrophoresis method for quantifying the binding of proteins to specific DNA regions: application to components of the *Escherichia coli* lactose operon regulatory system Nucleic Acids Res 1981 9 3047 3060 10.1093/nar/9.13.3047 6269071 PMC327330

[B19] Liu P Wood D Nester EW Phosphoenolpyruvate carboxykinase is an acid-induced, chromosomally encoded virulence factor in *Agrobacterium tumefaciens* J Bacteriol 2005 187 6039 6045 10.1128/JB.187.17.6039-6045.2005 16109945 PMC1196135

[B20] Cowie A Cheng J Sibley CD Fong Y Zaheer R Patten CL Morton RM Golding GB Finan TM An integrated approach to functional genomics: construction of a novel reporter gene fusion library for *Sinorhizobium meliloti* Appl Environ Microbiol 2006 72 7156 7167 10.1128/AEM.01397-06 16963549 PMC1636157

[B21] Caspi R Altman T Dreher K Fulcher CA Subhraveti P Keseler IM Kothari A Krummenacker M Latendresse M Mueller LA Ong Q Paley S Pujar A Shearer AG Travers M Weerasinghe D Zhang P Karp PD The MetaCyc database of metabolic pathways and enzymes and the BioCyc collection of pathway/genome databases Nucleic Acids Res 2012 40 D742 D753 10.1093/nar/gkr1014 22102576 PMC3245006

[B22] Kanehisa M Araki M Goto S Hattori M Hirakawa M Itoh M Katayama T Kawashima S Okuda S Tokimatsu T Yamanishi Y KEGG for linking genomes to life and the environment Nucleic Acids Res 2008 36 D480 D484 18077471 10.1093/nar/gkm882 PMC2238879

[B23] Jensen LJ Kuhn M Stark M Chaffron S Creevey C Muller J Doerks T Julien P Roth A Simonovic M Bork P von Mering C STRING 8--a global view on proteins and their functional interactions in 630 organisms Nucleic Acids Res 2009 37 D412 D416 10.1093/nar/gkn760 18940858 PMC2686466

[B24] Arias A Cerveñansky C Galactose metabolism in *Rhizobium meliloti* L5-30 J Bacteriol 1986 167 1092 1094 3745118 10.1128/jb.167.3.1092-1094.1986 PMC215990

[B25] Geddes BA Oresnik IJ Inability to catabolize galactose leads to increased ability to compete for nodule occupancy in *Sinorhizobium meliloti* J Bacteriol 2012 194 5044 5053 10.1128/JB.00982-12 22797764 PMC3430339

[B26] López-Lara IM Sohlenkamp C Geiger O Membrane lipids in plant-associated bacteria: their biosyntheses and possible functions Mol Plant Microbe Interact 2003 16 567 579 10.1094/MPMI.2003.16.7.567 12848422

[B27] Vences-Guzmán MA Geiger O Sohlenkamp C *Sinorhizobium meliloti* mutants deficient in phosphatidylserine decarboxylase accumulate phosphatidylserine and are strongly affected during symbiosis with alfalfa J Bacteriol 2008 190 6846 6856 10.1128/JB.00610-08 18708506 PMC2566212

[B28] BDGP Neural Network Promoter Prediction[http://www.fruitfly.org/seq_tools/promoter.html]

[B29] Barton LL Johnson GV Schitoskey K Wertz M Siderophore-mediated iron metabolism in growth and nitrogen fixation by alfalfa nodulated with *Rhizobium meliloti* J Plant Nutr 1996 19 1201 1210 10.1080/01904169609365191

[B30] O Cuív P Clarke P Lynch D O’connell M Identification of *rhtX* and *fptX*, novel genes encoding proteins that show homology and function in the utilization of the siderophores rhizobactin 1021 by *Sinorhizobium meliloti* and pyochelin by *Pseudomonas aeruginosa*, respectively J Bacteriol 2004 186 2996 3005 10.1128/JB.186.10.2996-3005.2004 15126460 PMC400637

[B31] Lynch D O’Brien J Welch T Clarke P Cuív PO Crosa JH O’Connell M Genetic organization of the region encoding regulation, biosynthesis, and transport of rhizobactin 1021, a siderophore produced by *Sinorhizobium meliloti* J Bacteriol 2001 183 2576 2585 10.1128/JB.183.8.2576-2585.2001 11274118 PMC95175

[B32] Viguier C O Cuív P Clarke P O’connell M RirA is the iron response regulator of the rhizobactin 1021 biosynthesis and transport genes in *Sinorhizobium meliloti* 2011 FEMS Microbiol Lett 2005 246 235 242 10.1016/j.femsle.2005.04.012 15899411

[B33] Chao T-C Buhrmester J Hansmeier N Puhler A Weidner S Role of the regulatory gene *rirA* in the transcriptional response of *Sinorhizobium meliloti* to iron limitation Appl Environ Microbiol 2005 71 5969 10.1128/AEM.71.10.5969-5982.2005 16204511 PMC1265945

[B34] Beck S Marlow VL Woodall K Doerrler WT James EK Ferguson GP The *Sinorhizobium meliloti* MsbA2 protein is essential for the legume symbiosis Microbiology (Reading, Engl) 2008 154 1258 1270 10.1099/mic.0.2007/014894-0 18375818

[B35] Griffitts JS Long SR A symbiotic mutant of *Sinorhizobium meliloti* reveals a novel genetic pathway involving succinoglycan biosynthetic functions Mol Microbiol 2008 67 1292 1306 10.1111/j.1365-2958.2008.06123.x 18284576

[B36] Jacob AI Adham SAI Capstick DS Clark SRD Spence T Charles TC Mutational analysis of the *Sinorhizobium meliloti* short-chain dehydrogenase/reductase family reveals substantial contribution to symbiosis and catabolic diversity Mol Plant Microbe Interact 2008 21 979 987 10.1094/MPMI-21-7-0979 18533838

[B37] Mauchline TH Fowler JE East AK Sartor AL Zaheer R Hosie AHF Poole PS Finan TM Mapping the *Sinorhizobium meliloti* 1021 solute-binding protein-dependent transportome Proc Natl Acad Sci USA 2006 103 17933 17938 10.1073/pnas.0606673103 17101990 PMC1635973

[B38] Chen H Teplitski M Robinson JB Rolfe BG Bauer WD Proteomic analysis of wild-type *Sinorhizobium meliloti* responses to N-acyl homoserine lactone quorum-sensing signals and the transition to stationary phase J Bacteriol 2003 185 5029 5036 10.1128/JB.185.17.5029-5036.2003 12923075 PMC180974

[B39] Clover RH Kieber J Signer ER Lipopolysaccharide mutants of *Rhizobium meliloti* are not defective in symbiosis J Bacteriol 1989 171 3961 3967 2738026 10.1128/jb.171.7.3961-3967.1989 PMC210148

[B40] Djordjevic SP Ridge RW Chen HC Redmond JW Batley M Rolfe BG Induction of pathogenic-like responses in the legume *Macroptilium atropurpureum* by a transposon-induced mutant of the fast-growing, broad-host-range *Rhizobium* strain NGR234 J Bacteriol 1988 170 1848 1857 2832384 10.1128/jb.170.4.1848-1857.1988 PMC211041

[B41] Newman JD Diebold RJ Schultz BW Noel KD Infection of soybean and pea nodules by *Rhizobium* spp. purine auxotrophs in the presence of 5-aminoimidazole-4-carboxamide riboside J Bacteriol 1994 176 3286 3294 8195084 10.1128/jb.176.11.3286-3294.1994 PMC205499

[B42] Noel KD Diebold RJ Cava JR Brink BA Rhizobial purine and pyrimidine auxotrophs: Nutrient supplementation, genetic analysis, and the symbiotic requirement for the novo purine biosynthesis Arch Microbiol 1988 149 499 506 10.1007/BF00446751

[B43] Danielli A Roncarati D Delany I Chiarini V Rappuoli R Scarlato V In vivo dissection of the *Helicobacter pylori* Fur regulatory circuit by genome-wide location analysis J Bacteriol 2006 188 4654 4662 10.1128/JB.00120-06 16788174 PMC1483005

[B44] Foreman DL Vanderlinde EM Bay DC Yost CK Characterization of a gene family of outer membrane proteins (*ropB*) in *Rhizobium leguminosarum* bv. viciae VF39SM and the role of the sensor kinase ChvG in their regulation J Bacteriol 2010 192 975 983 10.1128/JB.01140-09 20023026 PMC2812955

[B45] Dozot M Poncet S Nicolas C Copin R Bouraoui H Mazé A Deutscher J De Bolle X Letesson J-J Functional characterization of the incomplete phosphotransferase system (PTS) of the intracellular pathogen *Brucella melitensis* PLoS One 2010 5 e12679 10.1371/journal.pone.0012679 20844759 PMC2937029

[B46] Meade HM Long SR Ruvkun GB Brown SE Ausubel FM Physical and genetic characterization of symbiotic and auxotrophic mutants of *Rhizobium meliloti* induced by transposon Tn5 mutagenesis J Bacteriol 1982 149 114 122 6274841 10.1128/jb.149.1.114-122.1982 PMC216598

[B47] Galibert F Finan T Long S Pühler A Abola P Ampe F Barloy-Hubler F BARNETT M Becker A Boistard P Bothe G Boutry M Bowser L Buhrmester J Cadieu E Capela D Chain P Cowie A Davis R Dreano S Federspiel N FISHER R Gloux S Godrie T Goffeau A Golding B Gouzy J Gurjal M Hernández-Lucas I Hong A The composite genome of the legume symbiont *Sinorhizobium meliloti* Science 2001 293 668 672 10.1126/science.1060966 11474104

[B48] Hanahan D Studies on transformation of *Escherichia coli* with plasmids J Mol Biol 1983 166 557 580 10.1016/S0022-2836(83)80284-8 6345791

[B49] Studier FW Moffatt BA Use of bacteriophage T7 RNA polymerase to direct selective high-level expression of cloned genes J Mol Biol 1986 189 113 130 10.1016/0022-2836(86)90385-2 3537305

[B50] Yanisch-Perron C Vieira J Messing J Improved M13 phage cloning vectors and host strains: nucleotide sequences of the M13mp18 and pUC19 vectors Gene 1985 33 103 119 10.1016/0378-1119(85)90120-9 2985470

[B51] Kaniga K Delor I Cornelis GR A wide-host-range suicide vector for improving reverse genetics in gram-negative bacteria: inactivation of the *blaA* gene of *Yersinia enterocolitica* Gene 1991 109 137 141 10.1016/0378-1119(91)90599-7 1756974

[B52] Brody JR Kern SE History and principles of conductive media for standard DNA electrophoresis Anal Biochem 2004 333 1 13 10.1016/j.ab.2004.05.054 15351274

[B53] Ausubel FM Brent R Kingston RE Moore DD Seidman JG Smith JA Struhl K Short protocols in molecular biology 1992 2 New York: Greene Publishing Associates and John Wiley and Sons

[B54] Sambrook J Russell DW Molecular cloning: a laboratory manual, Vol 1-3 2001 3 Cold Spring Harbor, New York: Cold Spring Harbor Laboratory Press

[B55] Sinorhizobium meliloti 1021 [http://iant.toulouse.inra.fr/bacteria/annotation/cgi/rhime.cgi]

[B56] Finan TM Hartweig E Lemieux K Bergman K Walker GC Signer ER General transduction in *Rhizobium meliloti* J Bacteriol 1984 159 120 124 6330024 10.1128/jb.159.1.120-124.1984 PMC215601

