# Supplementary figures and images for: Members of the Sinorhizobium meliloti ChvI regulon identified by a DNA binding screen
Source: BMC Microbiol. 2013 Jun 13;13:132. doi: 10.1186/1471-2180-13-132 (PMC3687685; doi:10.1186/1471-2180-13-132)

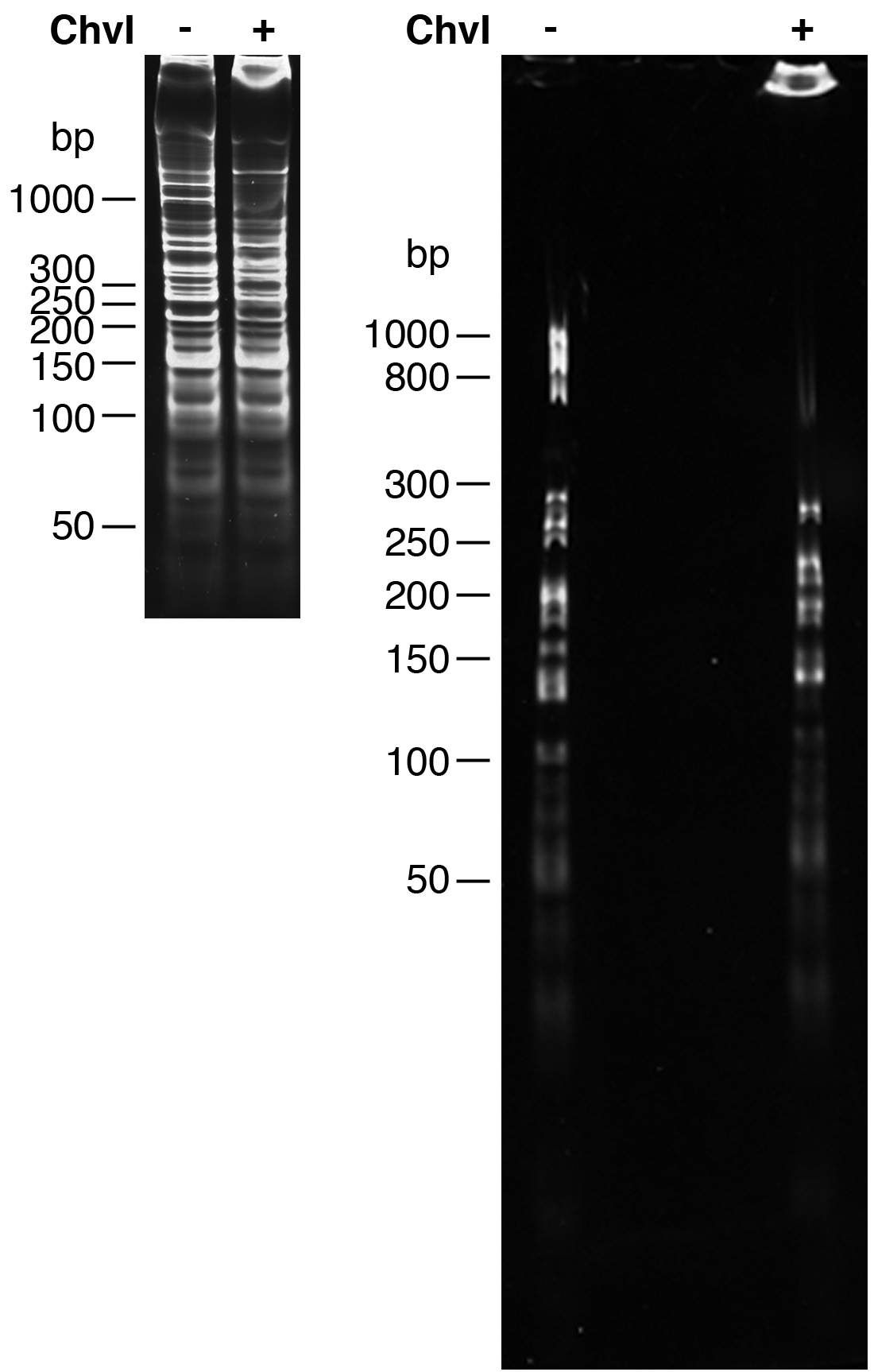

Supplement: Additional file 1 — Gel image of PD.EMSA to compare DNA shifts on 6-cm versus 14-cm 5% nondenaturing polyacrylamide gel and using SB buffer. Prior to the electrophoresis, the Bsp143I restricted pTC198 plasmid was incubated or not with the HisTag-ChvI protein. [file 1471-2180-13-132-S1.png]

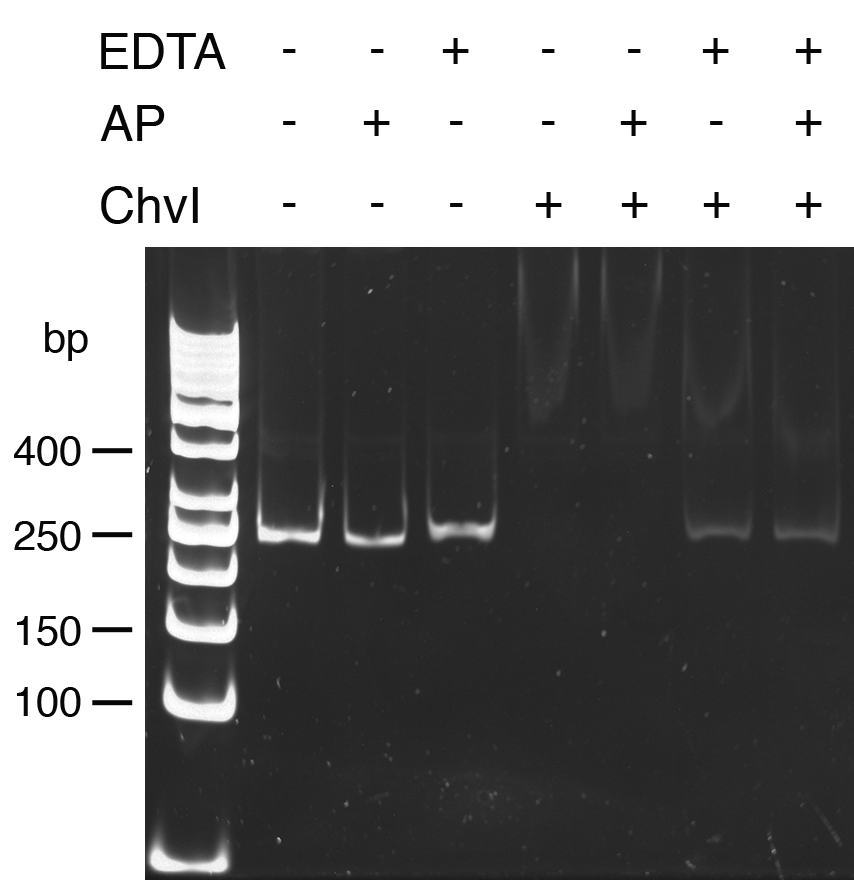

Supplement: Additional file 2 — Gel image of PD.EMSA to compare ChvI binding specificity in presence of EDTA or acetylphosphate. A 5% nondenaturing polyacrylamide gel made with TB buffer was used for the electrophoresis of the EcoRI-PstI double restricted pLB102 plasmid. The plasmid DNA was incubated or not with HisTag-ChvI protein in presence or not of EDTA and in presence or not of acetylphosphate (AP) prior to the electrophoresis. [file 1471-2180-13-132-S2.png]
